# The impact of previous history of malignant tumors/precancerous lesions on IVF/ICSI outcomes: a retrospective cohort study

**DOI:** 10.1080/07853890.2026.2654266

**Published:** 2026-05-08

**Authors:** Luyao Wang, Yifan Chu, Jiaxin Xie, Jiayun Chen, Yetan Liu, Shulin Yang, Peiwen Yang, Ying Xiao, Lei Jin, Ying Zhang, Jing Yue

**Affiliations:** ^a^ Reproductive Medicine and Genetics Center, Tongji Hospital, Tongji Medical College, Huazhong University of Science and Technology, Wuhan, China; ^b^ Department of Obstetrics and Gynecology, Tongji Hospital, Tongji Medical College, Huazhong University of Science and Technology, Wuhan, China; ^c^ Hubei Clinical Research Center for Reproductive Medicine, Shiyan, China; ^d^ Shiyan Key Laboratory of Reproduction and Genetics (Renmin Hospital, Hubei University of Medicine), Shiyan, China; ^e^ Reproductive Medicine Center, Renmin Hospital, Hubei University of Medicine, Shiyan, China

**Keywords:** Malignant tumors, precancerous lesions, in *vitro* fertilization, intracytoplasmic sperm injection, cumulative live birth rate

## Abstract

**Background:**

The incidence of malignant tumors/precancerous lesions is rising, yet their impact on pregnancy outcomes in women undergoing *in vitro* fertilization/intracytoplasmic sperm injection (IVF/ICSI) remains unclear.

**Methods:**

A single-center retrospective cohort study was conducted at the Reproductive Medicine and Genetics Center, Tongji Hospital, Tongji Medical College, Huazhong University of Science and Technology between January 2015 and December 2022. Propensity score matching (PSM) was applied to match the baseline data of the two groups, and the primary outcome were the conservative/optimistic cumulative live birth rates (CLBR) across multiple cycles.

**Results:**

A total of 1004 patients were enrolled, including 251 individuals in the malignant tumors/precancerous lesions group (363 oocyte retrieval cycles) and 753 PSM controls (895 oocyte retrieval cycles). Compared with the controls, patients with a history of malignant tumors/precancerous lesions exhibited significantly lower ovarian reserve and poorer embryo outcomes (all *p* < 0.05). Both conservative and optimistic CLBR were lower in the malignant tumors/precancerous lesions group across multiple cycles (*p* < 0.05). After multiple oocyte retrievals, the conservative CLBR for malignant tumors/precancerous lesions plateaued at around 53% after four cycles, while optimistic CLBR reached 70% after four cycles. Among patients with prior malignant tumors/precancerous lesions who completed follow-up, the overall recurrence rate after IVF/ICSI treatment was 7.03% with no adverse events.

**Conclusions:**

Patients with a previous history of malignant tumors/precancerous lesions demonstrated inferior live birth outcomes compared to controls. Multiple ovarian stimulations improved live birth opportunities without significantly increasing the risk of tumor recurrence.

## 1. Introduction

Recently, the global incidence of malignant tumors has been rising and shifting toward younger populations, posing a major public health concern [1,2]. With advances in diagnostic and therapeutic technologies, survival rates of cancer patients have improved significantly. However, anticancer treatments often cause pathological ovarian aging and increase the long-term risks of conditions such as osteoporosis, cardiovascular disease, and other metabolic disorders, all of which negatively affect patients’ quality of life [3,4].

Meanwhile, the incidence of precancerous lesions in the female reproductive system (e.g., cervical intraepithelial neoplasia [CIN], atypical endometrial hyperplasia [AEH]) is rising among younger populations, and the pathological changes and therapeutic interventions for their conditions may adversely affect fertility. Studies have suggested that CIN can disrupt cervical structure and mucus secretion, further impairing sperm migration and fertilization [5], and AEH patients may experience ovulatory dysfunction and reduced endometrial receptivity due to endometrial lesions, high-dose progesterone therapy, repeated uterine procedures, and comorbidities such as polycystic ovary syndrome, which all significantly increase infertility risks [6]. *In vitro* fertilization/intracytoplasmic sperm injection (IVF/ICSI), as the core assisted reproductive technologies (ART), provide critical clinical solutions for fulfilling the reproductive needs of such patients [7].

Findings remain inconsistent regarding the impact of a history of malignant tumors/precancerous lesions on the outcomes of IVF/ICSI. While Li et al. [8] and Turan et al. [9] suggested that cancer survivors exhibit similar oocyte yields and fertilization rates to the general infertile population, other evidence pointed to reduced pregnancy and live birth rates following IVF/ICSI, particularly among patients with gynecologic malignancies [10]. A recent meta-analysis indicated lower clinical pregnancy and live birth rates in cancer survivors relative to controls; however, significant heterogeneity among studies and inadequate adjustment for age-related confounders raise questions about the robustness of these conclusions [11]. Furthermore, limitations such as small sample sizes, variability in outcome measures, and unadjusted confounding factors constrain the generalizability of existing findings. Importantly, most published studies evaluate outcomes based on single IVF/ICSI cycles. Given the potential long-term reproductive impact of anticancer therapies, single-cycle protocols often yield low live birth probabilities, making multiple cycles necessary for many patients. Cumulative live birth rate (CLBR) [12], which integrates outcomes from all fresh and frozen-thawed embryo transfers following a single oocyte retrieval cycle, provides a comprehensive assessment of IVF/ICSI efficacy in cancer patients. However, research on CLBR in this population remains scarce.

For cancer survivors, achieving healthy offspring while ensuring personal safety is a key goal of collaborative management between reproductive medicine and oncology [13]. The potential risk of cancer recurrence associated with ovarian stimulation and oocyte retrieval remains controversial. Some studies have indicated that despite the hormone-dependent nature of cancers such as breast, cervical, and endometrial cancers, exogenous hormonal exposure during IVF/ICSI does not significantly increase recurrence risks[14,15]. However, cohort studies suggest that IVF/ICSI may increase the risk of ovarian cancer [16]. Regarding maternal-fetal outcomes, existing evidence links a history of malignancy to increased risks of pregnancy complications in IVF/ICSI cycles . Farland et al. [17], analyzing 662,630 deliveries in a national cohort, reported significantly higher risks of preterm birth and low birth weight in pregnant women with cancer history, though stratification by cancer type was lacking. Clarifying the impact of history of malignant tumors/precancerous lesions on pregnancy outcomes is crucial for developing individualized prenatal monitoring and intervention strategies.

Repeated ovarian stimulation may benefit young cancer survivors by improving CLBR. However, data on multi-cycle IVF/ICSI outcomes in this population remain limited. This study therefore investigates the clinical outcomes of IVF/ICSI in patients with a history of malignant tumors/precancerous lesions, aiming to inform clinical decision-making.

## 2. Study design and methods

### 2.1. Study population

This retrospective study included patients who underwent IVF/ICSI at the Reproductive Medicine Center of Tongji Hospital, Huazhong University of Science and Technology between January 2015 and December 2022. Patients were stratified into malignant/precancerous and control groups based on medical history. Exclusion criteria included (1) chromosomal abnormalities in either partner or preimplantation genetic testing (PGT); (2) testicular sperm aspiration (TESA)/percutaneous epididymal sperm aspiration (PESA)/microdissection testicular sperm extraction (mTESE); (3) donor oocyte/sperm cycles; (4) frozen sperm/oocyte or thawed oocyte cycles; (5) available embryos from other stimulation cycles; (6) recurrent spontaneous abortion (RSA), reproductive tract anomalies, autoimmune diseases, tuberculosis, etc.; (7) loss to follow-up or missing key data. Data on ovarian stimulation and embryo transfer were analyzed. Each IVF/ICSI cycle was followed for 2 years, with the final follow-up ending on December 31, 2024. Data were extracted from the center’s electronic medical records.

### 2.2. Ethical statement

This retrospective study was performed in compliance with relevant laws and institutional guidelines and was approved by the Ethics Committee of Tongji Hospital, Tongji Medical College, Huazhong University of Science and Technology (number: TJ-IRB202412050). The privacy rights of human subjects were observed, and written informed consent was obtained from each participant *via* a signed consent form.

### 2.3. Controlled ovarian hyperstimulation protocols

Controlled ovarian hyperstimulation (COH) protocols included gonadotropin-releasing hormone (GnRH) agonist, GnRH antagonist, progestin-primed ovarian stimulation (PPOS), and others (e.g., mild stimulation, natural cycles). Protocols were individualized based on age, body mass index (BMI), ovarian reserve, medical history, and prior treatment. Follicular growth was monitored *via* transvaginal ultrasound (TVS) every 2–4 days, with gonadotropin (Gn) doses adjusted according to serum hormone levels and follicular response. Triggering was performed with 0.25 mg recombinant human chorionic gonadotrophin (hCG) (Ovidrel^®^, Merck Serono) or 0.2 mg triptorelin acetate (Decapeptyl^®^, Ferring) combined with 2000 IU hCG (Livzon Group) when ≥ 2 follicles reached ≥ 18 mm or ≥ 3 follicles reached ≥ 17 mm.

### 2.4. Embryo culture and transfer

Oocyte retrieval was performed 36–38 h post-trigger under TVS guidance. Sperm samples were collected *via* masturbation on the day of retrieval. Fertilization *via* IVF/ICSI was performed based on semen parameters. Embryos were assessed for two pronuclei (2PN) 16–18 h post-fertilization and morphologically graded on Day 3. Fresh transfers of 1–2 Day 3 embryos or 1 Day 5/6 blastocysts were performed based on endometrial status. Remaining embryos were vitrified or cultured to blastocyst stage for freezing.

### 2.5. Endometrial preparation for frozen-thawed embryo transfer

Endometrial preparation protocols for frozen-thawed embryo transfer (FET) included artificial cycles, down-regulated artificial cycles, natural cycles, and ovulation induction. Endometrial transformation was initiated with progesterone (vaginal or intramuscular) when endometrial thickness reached ≥8 mm or post-ovulation. Embryo transfer occurred 3–6 days later.

### 2.6. Luteal support and follow-up

Luteal phase support for fresh cycles included oral dydrogesterone (Duphaston^®^, 20 mg/day) and vaginal progesterone gel (Crinone^®^, 90 mg/day). For FET cycles, oral estradiol valerate (Progynova^®^, 6 mg/day), dydrogesterone (20 mg/day), and vaginal progesterone capsules (Utrogestan^®^, 200 mg/day) were administered, with additional intramuscular progesterone (40 mg/day) if needed. Serum β-hCG was measured 12–14 days post-transfer. TVS confirmed intrauterine pregnancy at 4–6 weeks, with luteal support continued until 10–12 weeks of gestation. Telephone follow-ups tracked pregnancy outcomes, delivery, and neonatal status.

### 2.7. Outcome measures

Data extracted included malignant/precancerous history and follow-up, baseline characteristics and COH cycle details, which include total number of stimulated cycles, total number of Gn days, total amount of Gn, number of large follicles ≥ 14 mm on hCG day and estradiol (E_2_) level, COH protocol, insemination method, the number of oocytes obtained, metaphase II (MII) oocytes, 2PN embryos and available embryos (defined as the number of embryos for cryopreservation), the normal fertilization rate (2PN embryos/MII oocytes ×100%), blastocyst formation rate (blastocysts obtained/blastocysts cultured ×100%).

The primary outcome was the multi-cycle CLBR. Two approaches to calculating CLBR were documented: the multiple-cycle optimistic CLBR and the multiple-cycle conservative CLBR. The multiple-cycle optimistic CLBR assumes that patients who discontinued IVF/ICSI treatment during the process had the same probability of live birth as those who continued treatment in subsequent cycles. In contrast, the multiple-cycle conservative CLBR calculation assumes that patients who withdrew from IVF/ICSI treatment had a zero probability of live birth in subsequent cycles [18]. Secondary outcomes include (1) hCG positivity rate (number of hCG ≥ 10 IU/mL cycles/total number of transfer cycles ×100%); (2) clinical pregnancy rate (number of clinical pregnancy cycles/total number of transfer cycles ×100%); (3) miscarriage rate (number of miscarriage cycles/clinical pregnancy cycles ×100%); (4) ectopic pregnancy rate (number of ectopic pregnancy cycles/total number of transfer cycles ×100%); (5) live birth rate (number of live birth transfer cycles/total number of transfer cycles ×100%); (6) single cycle CLBR=(number of patients achieving their first live birth from a fresh or frozen cycle/number of COH cycles ×100%); (7) maternal-fetal outcomes including number of newborns, number of fetuses, mode of delivery, gestational week of delivery, newborn weight, macrosomia, low-birth-weight babies, preterm labor, gestational hypertension, gestational diabetes mellitus, placenta previa, abruptio placentae, premature rupture of membranes, neonatal jaundice, and birth defects.

Clinical pregnancy was defined as intrauterine gestational sac visible by TVS at 4–6 weeks post-transfer; miscarriage was defined as termination of pregnancy at less than 28 weeks of gestation with a fetal weight of <1000 g; preterm delivery was defined as delivery of at least one live infant between 28 and 36^+6 ^weeks of gestation; and live birth was defined as delivery at ≥ 28 weeks of gestation in which the neonate possessed respiration, heartbeat, umbilical arterial pulsation, or random muscle contractions as any of the vital signs.

### 2.8. Statistical analysis

Data collected were analyzed by R 4.3.0 and SPSS 27 (IBM, Chicago, IL) for propensity score matching (PSM, 1:3 ratio, caliper = 0.2). Continuous variables were expressed as mean ± SD or median (IQR) and compared *via* t-test/Wilcoxon test. Categorical variables were analyzed *via* chi-square/Fisher’s exact test. Kaplan-Meier curves and log-rank tests compared CLBR between groups. A two-sided *p* < 0.05 indicated significance.

## 3. Results

### 3.1. Impact of history of malignant tumors/precancerous lesions on IVF/ICSI outcomes

**Figure 1.**
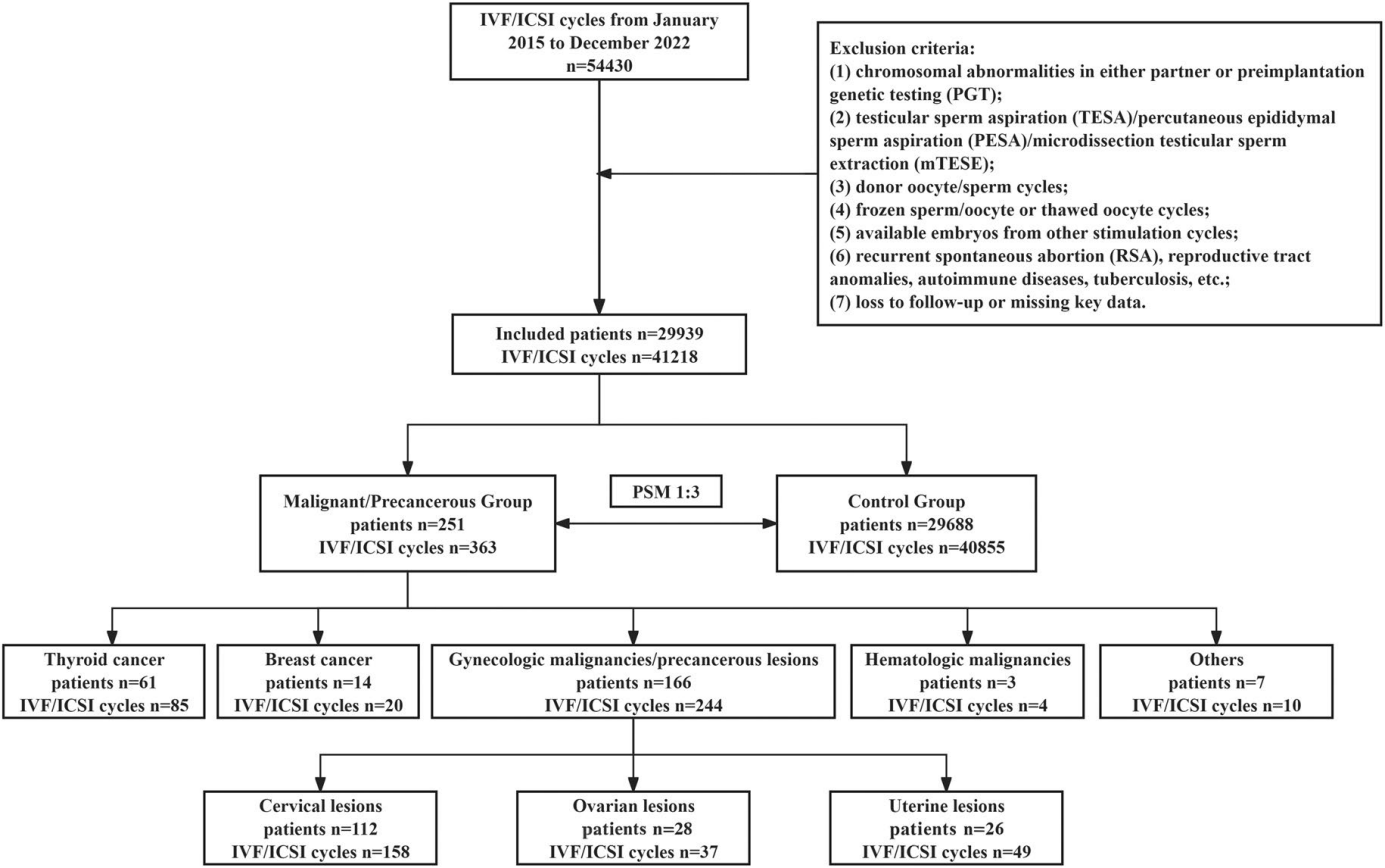
Flowchart of this study.

**Figure 2.**
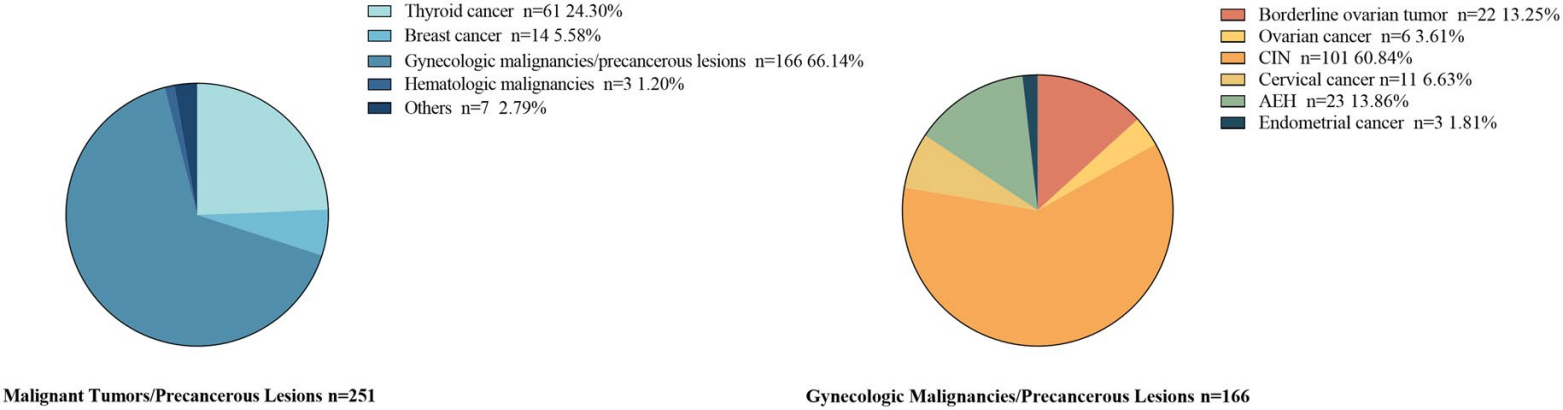
Distribution of malignant tumors/precancerous lesions by type.

Of 54,430 IVF/ICSI cycles screened, 29,939 patients (41,218 cycles) were included: 251 patients (363 cycles) with malignant tumors/precancerous lesions histories and 29,688 controls (40,855 cycles). The malignant tumors/precancerous lesions group included thyroid cancer (61 cases), breast cancer (14), hematologic malignancies (3), gynecologic malignancy/precancerous lesions (166), and others (7). Gynecologic cases comprised cervical lesions (112), uterine lesions (26), and ovarian lesions (28) (Figures 1, 2).

#### 3.1.1. Baseline and COH cycle characteristics

After performing PSM based on the patients’ age, BMI, type of infertility, and duration of infertility, the malignant/precancerous group had lower antral follicle count (AFC) [9.0 (5.5–12.0) vs. 11.0 (7.0–17.0), *p* < 0.001] and anti-Müllerian hormone (AMH) levels [2.48 (1.34–4.35) vs. 3.13 (1.74–5.62) ng/mL, *p* < 0.001], with a higher proportion of ovarian insufficiency-related infertility (42.63% vs. 25.50%, *p* < 0.001). Other baseline parameters showed no significant differences (*p* > 0.05) (Table 1).

**Table 1.** Baseline characteristics between two groups.

Variable	Malignant Tumors/Precancerous Lesions Group (*n* = 251)	Control Group (*n* = 753)	Statistic	*p*
Age (years), M (Q₁, Q₃)	32.0 (29.0–35.0)	32.0 (29.0–35.0)	Z=-1.22	0.222
BMI (kg/m²), M (Q₁, Q₃)	21.72 (20.13–23.91)	21.78 (19.95–24.03)	Z=-0.29	0.773
bFSH (mIU/mL), M (Q₁, Q₃)	7.29 (6.13–8.89)	7.30 (6.20–8.88)	Z=-0.34	0.731
AFC (n), M (Q₁, Q₃)	9.0 (5.5–12.0)	11.0 (7.0–17.0)	Z=-5.13	<0.001*
AMH (ng/mL), M (Q₁, Q₃)	2.48 (1.34–4.35)	3.13 (1.74–5.62)	Z=-3.78	<0.001*
Infertility duration (years), M (Q₁, Q₃)	2.5 (1.0–4.0)	3.0 (2.0–5.0)	Z=-1.60	0.109
Infertility type, n (%)			χ²=0.01	0.909
Primary	163 (64.94)	492 (65.34)		
Secondary	88 (35.06)	261 (34.66)		
Cause of Infertility, n (%)				
DOR	107 (42.63)	192 (25.50)	χ²=26.42	<0.001*
Ovulatory disorders	16 (6.37)	34 (4.52)	χ²=1.38	0.241
Tubal/pelvic factors	117 (46.61)	377 (50.07)	χ²=0.90	0.343
Uterine factors	54 (21.51)	148 (19.65)	χ²=0.40	0.525
Endometriosis	27 (10.76)	79 (10.49)	χ²=0.01	0.906
Male factors	64 (25.50)	194 (25.76)	χ²=0.01	0.934
Unexplained	12 (4.78)	51 (6.77)	χ²=1.27	0.260

Values are presented as median (25th percentile, 75th percentile) or proportion (%).

*p < 0.05.

BMI: body mass index; bFSH: basal follicle-stimulating hormone; AFC: antral follicle count; AMH: anti-Müllerian hormone; DOR: diminished ovarian reserve.

#### 3.1.2. COH and embryo transfer outcomes

The malignant/precancerous group required fewer Gn days, lower Gn doses, fewer follicles ≥ 14 mm on trigger day, and lower E_2_ levels (*p* < 0.05). Antagonist protocol was more common in the study group, while agonist protocol dominated the controls. The study group also had fewer oocytes retrieved, MII oocytes, 2PN embryos, fertilization rates, and usable embryos (*p* < 0.05). Transfer cycle characteristics (embryo type, number, endometrial preparation) showed no significant differences (*p* > 0.05) (Table 2).

**Table 2.** COH outcomes and embryo transfer data between two groups.

Variable	Malignant Tumors/Precancerous Lesions Group (*n* = 251)	Control Group (*n* = 753)	Statistic	*p*
COH cycles	363	895	—	—
Gn days, M (Q₁, Q₃)	9.0 (8.0–11.0)	10.0 (9.0–11.0)	Z = −3.23	0.001*
Total Gn dose (IU), M (Q₁, Q₃)	2220.00 (1318.50–2943.50)	2400.00 (1800.00–2925.00)	Z = −3.57	<0.001*
Follicles ≥ 14 mm on hCG day, M (Q₁, Q₃)	7.0 (4.0–11.0)	9.0 (5.0–12.0)	Z = −4.78	<0.001*
E_2_ on hCG day (pg/ml), M (Q₁, Q₃)	1530.00 (892.50–2560.50)	2114.00 (1320.00–3400.00)	Z = −6.48	<0.001*
COH protocol, n (%)			χ² = 45.27	<0.001*
GnRH agonist	109 (30.03)	420 (46.93)		
GnRH antagonist	150 (41.32)	343 (38.32)		
PPOS	84 (23.14)	111 (12.40)		
Others	20 (5.51)	21 (2.35)		
Fertilization method, n (%)			χ² = 0.68	0.411
IVF	234 (66.48)	609 (68.89)		
ICSI	118 (33.52)	275 (31.15)		
Total oocytes retrieved, M (Q₁, Q₃)	8.0 (4.0–14.0)	10.0 (6.0–15.0)	Z = −4.43	<0.001*
MII oocytes, M (Q₁, Q₃)	7.0 (3.0–11.0)	8.0 (5.0–13.0)	Z = −4.42	<0.001*
2PN embryos, M (Q₁, Q₃)	5.0 (2.0–8.0)	6.0 (3.0–9.0)	Z = −4.77	<0.001*
Fertilization rate, % (n)	57.38 (1959/3414)	60.47 (6025/9963)	χ² = 10.12	0.001*
Blastocyst formation rate, % (n)	64.72 (985/1522)	66.76 (3322/4976)	χ² = 2.18	0.140
Available embryos, M (Q₁, Q₃)	2.0 (1.0–4.0)	3.0 (2.0–5.0)	Z = −5.14	<0.001*
Transfer cycles	413	1171	—	—
Transfer cycle type, % (n)			χ² = 54.61	<0.001*
Fresh cycles only	19.83 (72/363)	31.28 (280/895)		
Thawed cycles only	46.28 (168/363)	39.33 (352/895)		
Fresh + thawed cycles	11.57 (42/363)	19.55 (175/895)		
No transfer	22.31 (81/363)	9.83 (88/895)		
Embryos transferred	517	1486	—	—
Embryo type transferred, % (n)			χ² = 1.35	0.245
Cleavage-stage	52.22 (270/517)	55.18 (820/1486)		
Blastocyst	47.78 (247/517)	44.82 (666/1486)		
Number of embryos transferred, % (n)			χ² = 0.46	0.496
Single	74.82 (309/413)	73.14 (856/1171)		
Double	25.18 (104/413)	26.85 (315/1171)		
Endometrial preparation for thawed cycles, % (n)			χ² = 3.74	0.291
Artificial cycle	69.57 (208/299)	74.20 (532/716)		
Natural cycle	9.36 (28/299)	9.62 (69/716)		
Down-regulated + artificial cycle	18.39 (55/299)	13.83 (99/716)		
Stimulated cycle	2.68 (8/299)	2.23 (16/716)		

Values are presented as median (25th percentile, 75th percentile) or proportion (%).

*p < 0.05.

COH: controlled ovarian hyperstimulation; Gn: gonadotrophin; hCG: human chorionic gonadotrophin; PPOS: progestin-primed ovarian stimulation; IVF: *in vitro* fertilization; ICSI: intracytoplasmic sperm injection; MII: metaphase II; 2PN: two pronucleus.

#### 3.1.3. Pregnancy, maternal-fetal outcomes, and CLBR

The malignant tumors/precancerous lesions group had lower hCG positivity (48.18% vs. 53.80%), clinical pregnancy (42.37% vs. 48.08%), live birth (32.69% vs. 39.97%), and shorter gestational age [38.3 (37.2–39.1) vs. 38.8 (37.9–39.5) weeks], with higher rates of low birth weight (21.92% vs. 11.18%) and preterm birth (22.22% vs. 14.74%) (all *p* < 0.05). Miscarriage and ectopic pregnancy rates were comparable (*p* > 0.05) (Table 3).

**Table 3.** Pregnancy and maternal-fetal outcomes between two groups.

Variable	Malignant Tumors/Precancerous Lesions Group (*n* = 251)	Control Group (*n* = 753)	Statistic	*p*
Pregnancy outcomes, % (n)				
hCG positivity rate	48.18 (199/413)	53.80 (630/1171)	χ² = 3.86	0.049*
Clinical pregnancy rate	42.37 (175/413)	48.08 (563/1171)	χ² = 3.99	0.046*
Miscarriage rate	22.86 (40/175)	16.87 (95/563)	χ² = 3.20	0.074
Ectopic pregnancy rate	2.42 (1/413)	0.18 (2/1171)	—	1.000
Live birth rate	32.69 (135/413)	39.97 (468/1171)	χ² = 6.86	0.009*
Maternal-fetal outcomes				
Number of neonates	146	510	—	—
Fetal number, % (n)			χ² = 0.09	0.765
Singleton	91.85 (124/135)	91.03 (426/468)		
Twin	8.15 (11/135)	8.97 (42/468)		
Delivery mode, % (n)			χ² = 0.37	0.542
Vaginal delivery	16.30 (22/135)	18.59 (87/468)		
Cesarean section	83.70 (113/135)	81.41 (381/468)		
Gestational age (weeks), M (Q₁, Q₃)	38.3 (37.2–39.1)	38.8 (37.9–39.5)	Z = −3.67	<0.001*
Neonatal weight (kg), M (Q₁, Q₃)	3.15 (2.69–3.50)	3.20 (2.83–3.50)	Z = −1.36	0.174
Macrosomia, % (n)	2.74 (4/146)	3.33 (17/510)	χ² = 0.01	0.926
Low birth weight, % (n)	21.92 (32/146)	11.18 (57/510)	χ² = 11.17	<0.001*
Preterm birth, % (n)	22.22 (30/135)	14.74 (69/468)	χ² = 4.27	0.039*
Gestational hypertension, % (n)	3.70 (5/135)	2.99 (14/468)	χ² = 0.02	0.890
Gestational diabetes, % (n)	5.93 (8/135)	4.27 (20/468)	χ² = 0.65	0.422
Placenta previa, % (n)	2.22 (3/135)	1.71 (8/468)	χ² = 0.00	0.978
Premature rupture of membranes, % (n)	2.96 (4/135)	2.14 (10/468)	χ² = 0.06	0.812
Neonatal jaundice, % (n)	2.74 (4/146)	4.71 (24/510)	χ² = 1.07	0.300
Neonatal birth defects, % (n)	2.74 (4/146)	2.35 (12/510)	χ² = 0.00	1.000

Values are presented as median (25th percentile, 75th percentile) or proportion (%).

*p < 0.05.

hCG: human chorionic gonadotrophin.

**Figure 3.**
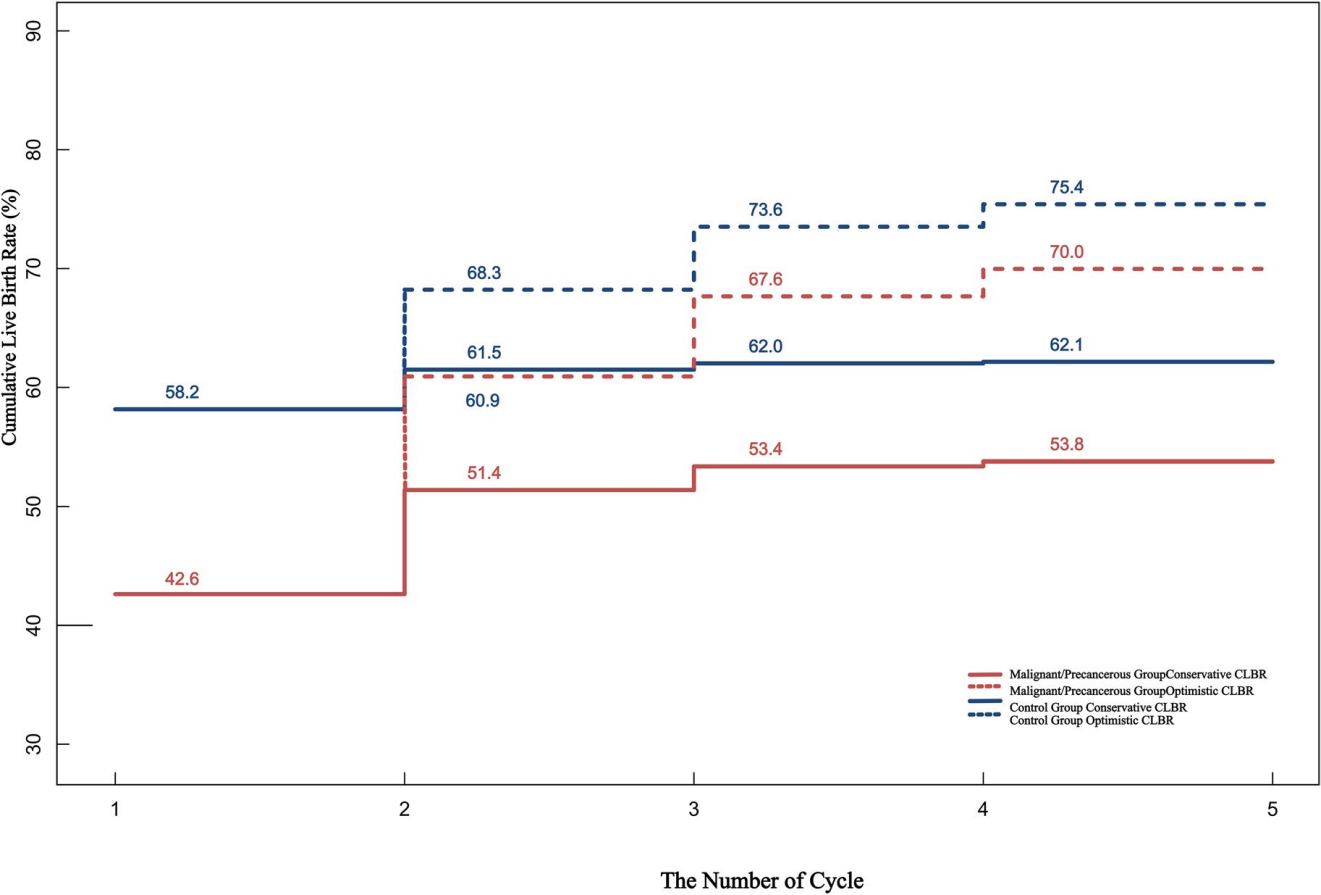
Cumulative live birth rate between two groups.

As shown in Table 4, after 3–4 cycles, the CLBR plateaued. The study group’s conservative and optimistic CLBR after 4 cycles were 53.8% and 70.0%, respectively, versus 62.1% and 75.4% in controls (log-rank *p* < 0.05) (Table 4, Figure 3).

**Table 4.** Multi-cycle pregnancy outcomes between two groups.

					Multi-Cycle CLBR (%)	
Group	Number of Patients	Oocyte Retrieval Cycles	Live Birth	Single-Cycle CLBR (%)	Conservative CLBR% (95% CI)	Optimistic CLBR% (95% CI)
Malignant Tumors/Precancerous Lesions Group						
Cycle 1	251	246	107	42.6	42.6 (36.5–48.7)	42.6 (36.5–48.7)
Cycle 2	69	66	22	31.9	51.4 (45.2–57.6)	60.9 (54.9–67.0)
Cycle 3	29	27	5	17.2	53.4 (47.2–59.6)	67.6 (61.9–73.5)
≥4 Cycles	14	13	1	7.1	53.8 (47.6–60.0)	70.0 (64.3–75.7)
Control Group						
Cycle 1	753	746	438	58.2	58.2 (54.6–61.7)	58.2 (54.6–61.7)
Cycle 2	104	100	25	24.0	61.5 (58.0–65.0)	68.3 (64.9–71.6)
Cycle 3	24	23	4	16.7	62.0 (58.6–65.5)	73.6 (70.4–76.7)
≥4 Cycles	14	14	1	7.1	62.1 (58.7–65.6)	75.4 (72.3–78.5)

CLBR: cumulative live birth rate; CI: confidence interval.

Given that thyroid cancer and cervical lesions were the most common malignant tumors/precancerous lesions in this study, subgroup analyses were conducted for 61 thyroid cancer patients and 112 cervical lesions patients using the same PSM method.

#### 3.2. Impact of history of thyroid cancer on IVF/ICSI outcomes

##### 3.2.1. Baseline and COH cycle characteristics

As shown in Table S1, the thyroid cancer subgroup showed no significant differences in baseline characteristics compared to its matched controls. In contrast, the cervical lesions subgroup had significantly lower AFC and AMH levels, as well as a higher proportion of DOR-related infertility (*p* < 0.05), while other baseline parameters did not differ significantly (*p* > 0.05).

#### 3.2.2. COH outcomes and embryo transfer data between subgroups

As shown in Table S2, compared to their respective control groups, the thyroid cancer subgroup exhibited significantly lower E_2_ levels on hCG day and a higher proportion of ICSI-assisted reproduction (*p* < 0.05). The cervical lesion subgroup showed significantly fewer follicles ≥ 14 mm on hCG day, lower E2 levels on hCG day, fewer oocytes retrieved, fewer MII oocytes, fewer 2PN embryos, and fewer usable embryos (*p* < 0.05). In transfer data, both the thyroid cancer and cervical lesions subgroups had a significantly higher proportion of thawed cycles compared to their controls (*p* = 0.007), while other transfer parameters showed no significant differences (*p* > 0.05).

#### 3.2.3. Pregnancy, maternal-fetal outcomes, and CLBR

The pregnancy and maternal-fetal outcomes between groups are summarized in Table S3. The thyroid cancer group showed similar pregnancy and maternal-fetal outcomes to the control group, with no statistically significant differences (*p* > 0.05). In contrast, the cervical lesions group had a lower clinical pregnancy rate (34.78% vs. 48.08%), lower live birth rate (26.09% vs. 39.42%), reduced gestational age at delivery [38.2 (36.8–39.1) vs. 38.9 (37.9–39.5) weeks], and higher rates of lower birth weight infants (28.57% vs.15.11%) and preterm birth (27.78% vs. 15.61%), with all these differences being statistically significant (*p* < 0.05). The remaining pregnancy and maternal-fetal outcomes showed no significant differences between groups (*p* > 0.05).

As shown in Table S4, the live birth rate curves for the thyroid cancer group, cervical lesions group, and their respective control groups plateaued after 3–4 treatment cycles (Figure S1). In the thyroid cancer group, the conservative CLBR reached 60.7% and the optimistic CLBR reached 72.6% after four IVF/ICSI cycles, while the control group achieved a conservative CLBR of 59.6% and an optimistic CLBR of 74.8%. For the cervical lesions group, the conservative CLBR was 48.2% and the optimistic CLBR was 63.1% after four cycles, compared to the control group’s conservative CLBR of 61.0% and optimistic CLBR of 69.6%. Log-rank tests revealed no significant differences in conservative or optimistic CLBR between the thyroid cancer group and its controls (*p* > 0.05), whereas the cervical lesions group showed statistically significant differences in both CLBR measures compared to its controls (*p* < 0.05).

### 3.3. Follow-up outcome analysis of patients with malignant tumors/precancerous lesions

Long-term follow-up of patients with a history of prior malignancy/precancerous lesions who underwent IVF/ICSI-assisted conception was performed from the first IVF/ICSI treatment to December 2024, as shown in Table 5. Of the 185 patients who actually completed follow-up, with a median follow-up of 50 months, the overall recurrence rate of malignant tumors/precancerous lesions was 7.03% (13/185). Among them, one patient with ovarian borderline tumor experienced recurrence after IVF/ICSI, and the recurrence rate was 4.55% (1/22); nine patients with CIN experienced recurrence after IVF/ICSI, and the recurrence rate was 12.50% (9/72), and three patients with AEH experienced recurrence after IVF/ICSI, and the recurrence rate was 15.79% and no cases of recurrence of malignant tumors/precancerous lesions were observed in the remaining AEH patients who completed follow-up.

**Table 5.** Information and follow-up outcomes of patients with malignant tumors/precancerous lesions.

Category	Number of Cases	Treatment Modality	Follow-up (Completed/Total)	Follow-up Time (Months), M(Q₁, Q₃)	Follow-up Outcomes
Thyroid cancer	61	Surgery ± I-131 therapy	42/61	42.5 (32.0, 64.0)	No recurrence
Breast cancer	14	Chemotherapy ± radiotherapy ± endocrine therapy ± surgery	12/14	55.5 (34.3, 75.5)	No recurrence
Gynecologic malignancies/precancerous lesions					
Borderline ovarian tumor	22	Surgery ± chemotherapy	15/22	47.0 (39.5, 62.0)	1 recurrence
Ovarian cancer	6	Surgery ± chemoradiotherapy	5/6	41.0 (35.0, 50.0)	No recurrence
CIN	101	Expectant management ± cervical conization	72/101	57.0 (37.0, 79.5)	9 recurrences
Cervical cancer	11	Cervical conization ± chemoradiotherapy	8/11	42.5 (34.5, 55.5)	No recurrence
AEH	23	Fertility-sparing treatments	19/23	45.0 (39.0, 63.0)	3 recurrences
Endometrial cancer	3	Fertility-sparing treatments	3/3	72.0 (66.5, 76.5)	No recurrence
Hematologic malignancies	3	Chemotherapy	3/3	89.0 (60.5, 100.0)	No recurrence
Others	7	Chemoradiotherapy ± surgery	6/7	70.0 (47.3, 84.5)	No recurrence

CIN: Cervical intraepithelial neoplasia; AEH: Atypical endometrial hyperplasia.

Fertility-sparing treatments: oral progestin therapy, GnRH agonists, levonorgestrel-releasing intrauterine system, aromatase inhibitors, metformin, and combination therapies. Others: gastric cancer, lung cancer, nasopharyngeal carcinoma, craniopharyngioma, and neuroblastoma.

## 4. Discussion

Although IVF/ICSI offers a promising solution for these patients with a history of malignant tumors/precancerous lesions, studies on the overall reproductive outcomes remain limited. Through a retrospective cohort study, we systematically compared IVF/ICSI outcomes between patients with prior malignant tumors/precancerous lesions histories and controls and our results demonstrated that patients with malignant tumors/precancerous lesions had significantly lower conservative and optimistic CLBR than controls. However, conservative CLBR plateaued after multiple oocyte retrievals, stabilizing at approximately 53% after four retrievals, while optimistic CLBR continued to rise, reaching approximately 70% after four retrievals. Consistent with prior research, patients with malignant tumors/precancerous lesions histories often exhibit diminished ovarian reserve due to anticancer therapies, yet multi-cycle IVF/ICSI interventions can still yield relatively satisfactory outcomes. These findings underscore the importance of individualized treatment strategies, suggesting that extending treatment cycles may optimize reproductive outcomes.

### 4.1. Impact of thyroid cancer history on IVF/ICSI outcomes

Thyroid cancer is one of the most common malignancies in reproductive-aged women, with a 5-year survival rate exceeding 90%. While thyroidectomy (total/partial) and radioactive iodine (I-131) therapy are standard treatments, their effects on fertility remain debated. Thyroid cancer patients often require long-term thyroid hormone replacement to maintain metabolic homeostasis. Secondary thyroid dysfunction may disrupt the hypothalamic-pituitary-ovarian axis, leading to menstrual irregularities, diminished ovarian reserve, ovulatory disorders, and adverse pregnancy outcomes. Our study found no significant differences in ovarian function, COH outcomes, pregnancy outcomes, or maternal-fetal outcomes between thyroid cancer patients and controls. Multi-cycle CLBR analyses further revealed trends comparable to matched infertile controls, indicating that IVF/ICSI outcomes in thyroid cancer patients are more favorable than in other malignancies. Existing studies also support that thyroid cancer history does not compromise IVF/ICSI efficacy or obstetric safety, reinforcing its feasibility for this population [19,20].

The reproductive safety of thyroid cancer treatments requires careful consideration. Partial thyroidectomy has been associated with 7-fold higher clinical pregnancy rates and 6-fold higher live birth rates compared to total thyroidectomy [21]. This difference may arise because total thyroidectomy can disrupt the hypothalamic-pituitary-thyroid axis, which in turn may disturb gonadotropin pulsatility, calcium regulation, and vitamin D metabolism [21]. Additionally, postoperative thyrotropin suppression may imbalance Th1/Th2 cytokine ratios, reduce endometrial integrin αvβ3 and leukemia inhibitory factor expression, and impair embryo implantation [22]. Furthermore, radioactive I-131 therapy can induce DNA double-strand breaks in granulosa cells and mitochondrial dysfunction and cause dose-dependent ovarian damage through β/γ radiation [23,24]. AMH levels have also been shown to drop by 32% within 12 months after treatment [24]. Cohort data further associate I-131 exposure with lower clinical pregnancy and live birth rates [25], with real-world analyses showing increased risk of congenital anomalies and miscarriages in pregnancies conceived within 6 months after I-131 treatment, but not in those conceived 6 months or later [26]. These findings are consistent with Huang et al. who reported that initiating IVF/ICSI at least 6 months after I-131 does not compromise outcomes [21]. Thus, optimizing fertility outcomes in thyroid cancer patients requires balancing individualized treatment modalities (surgical extent, radiation dose) and ART timing.

### 4.2. Impact of cervical lesion history on IVF/ICSI outcomes

In patients with cervical lesions (e.g., CIN or early-stage cervical cancer), our study demonstrated significantly reduced ovarian reserve, oocyte yield, embryo laboratory outcomes, clinical pregnancy rates, live birth rates, and CLBR compared to controls, consistent with findings by Lin et al. [10] and Yang et al. [27]. Although cervical treatments such as conization primarily target localized lesions without direct ovarian involvement, the observed reproductive impairment may stem from human papillomavirus (HPV)-related mechanisms. Growing evidence links HPV infection to female infertility, with cohort studies reporting a 39% higher infertility risk in HPV-positive women (aHR: 1.39, 95% CI: 1.19–1.63) [28]; these women also exhibit lower IVF pregnancy rates (23.0% vs. 57.0%, *p* < 0.05) and reduced AMH levels [29,30]. Meanwhile, over 99% of cervical lesions are caused by high-risk HPV (HR-HPV), which can trigger chronic inflammation, oxidative stress, and pelvic immune dysregulation [31]. These factors may further disrupt oocyte meiosis, damage granulosa cells, impair embryo development (e.g., 25.9% reduced blastocyst formation with HPV16 exposure in mice) [32], and compromise endometrial receptivity, as HR-HPV has been detected in 26% of endometriosis-associated infertile endometria [33]. Regrettably, the CIN cases in this study were not further stratified by grade, which may affect the precise assessment of the impact of cervical lesions. Despite shifting HPV infection peaks to younger ages, the differential impacts of HR-HPV versus low-risk HPV on ovarian reserve, embryogenesis, and endometrial function remain unclear, necessitating large-scale cohort studies with viral genotyping. Furthermore, transcriptional HPV integration, a key driver of cervical carcinogenesis, promotes immune evasion *via* tumor cell reprogramming [34]. Future research should explore HPV integration’s predictive value for ART outcomes and assess antiviral therapies in improving success rates.

Notably, cervical lesion patients in our study had higher preterm birth rates, potentially linked to treatment-induced cervical incompetence. While recent studies suggest cervical treatments for CIN2/3 do not significantly affect IVF/ICSI outcomes [35], delayed ART (>1 year post-diagnosis) may reduce pregnancy rates [27]. However, the impact of cervical interventions on maternal-fetal outcomes remains understudied. Preemptive preconception cervical assessment and targeted interventions are essential to minimize obstetric risks in this population.

### 4.3. Long-term safety of IVF/ICSI in malignant tumors/precancerous lesions survivors

When treating infertile women of reproductive age with a history of malignant tumors/precancerous lesions, it is necessary to further clarify the potential and long-term safety impacts of the lesion types and treatments on female reproductive function. Although the safety of IVF/ICSI technology is widely recognized, it remains unresolved whether the use of exogenous gonadotropin stimulation during COH and the elevated hormone levels during pregnancy may lead to recurrence or abnormal pathological conditions in certain specific hormone-dependent tumors. Our follow-up data revealed a 7.03% overall recurrence rate in malignant tumors/precancerous lesions patients post-IVF/ICSI, with no deaths reported. Recurrence rates and survival outcomes aligned with expected prognoses, supporting the safety of ART in cured patients. However, large-scale cohort studies are needed for validation. For gynecologic malignancy/precancer survivors, current guidelines recommend close post-conception surveillance [36] and definitive surgery for high-risk cases with persistent/recurrent lesions (e.g., HPV persistence, positive conization margins, or AEH progression) [37–39]. Clinicians must balance oncologic safety with reproductive goals, employing multidisciplinary evaluations, thorough patient counseling, and vigilant pregnancy monitoring.

In the fertility decision-making for patients with malignant tumors/precancerous lesions, fertility preservation (FP) has emerged as a critical focus in the intersecting fields of reproductive medicine and oncology, which primarily include embryo/oocyte cryopreservation, ovarian tissue cryopreservation and *in vitro* maturation [40,41]. Concurrently, the timely initiation of assisted reproductive interventions following the completion of anticancer therapy is equally vital. It is crucial to emphasize that a comprehensive approach should involve both partners. For male patients with a history of malignancy, anticancer therapies can also impair spermatogenesis, leading to conditions such as oligospermia or even cryptic sperm defects—subtle functional abnormalities that may not be detected by standard semen analysis but can contribute to fertilization failure or poor embryo quality [42]. Of note, beyond conventional medical decisions, emerging evidence suggests that adjuvant nutritional interventions, such as myo-inositol and alpha-lipoic acid [43,44], may also play a beneficial role in the management of infertility among specific cancer survivors.

This study has several limitations. As a single-center retrospective study, the study sample was limited and encompassed diverse tumor types with heterogeneous pathological features, which may limit the generalizability of the conclusions. Additionally, the inability to obtain pre-disease baseline reproductive function data makes it difficult to establish causality. Furthermore, due to limited access to medical records, we did not specifically evaluate the impact of different chemotherapeutic agents or radiation doses for malignant tumors/precancerous lesions conditions on IVF/ICSI outcomes and natural pregnancy, nor did we conduct long-term follow-up on the development and genetic risks of offspring. Larger multicenter studies and extended follow-up data are needed to further explore the safety and efficacy in these aspects.

## Conclusion

This study compared IVF/ICSI outcomes between patients with a history of malignant tumors/precancerous lesions and matched control subjects. It confirmed that a history of malignant tumors/precancerous lesions may significantly impair reproductive function, leading to decreased live birth rates and cumulative live birth rates. However, multiple ovarian stimulation cycles can increase the chance of live birth, notably in patients with a history of cervical lesions. For patients with malignant tumors/precancerous lesions who have fertility desires, regular ovarian function monitoring is recommended. Before undergoing IVF/ICSI, they should receive comprehensive examinations and multidisciplinary consultations to select the optimal timing and method for assisted reproduction, ensuring the well-being of both the mother and offspring.

## Supplementary Material

Supplementary_materials complete.docx

## Data Availability

The datasets used and/or analysed during the current study are available from the corresponding author on reasonable request.
