# Supplementary material for: The impact of previous history of malignant tumors/precancerous lesions on IVF/ICSI outcomes: a retrospective cohort study
Source: Ann Med. 2026 May 8;58(1):2654266. doi: 10.1080/07853890.2026.2654266 (PMC13159594; doi:10.1080/07853890.2026.2654266)
Supplement: Supplementary_materials complete.docx [file IANN_A_2654266_SM9736.docx]

| **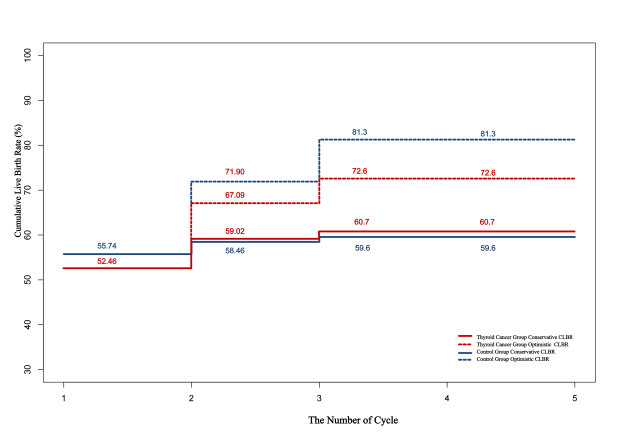** | **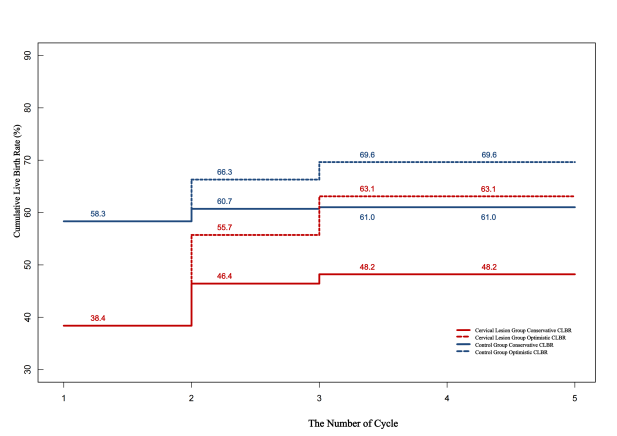** |
| --- | --- |

**Supplementary Figure 1 Cumulative live birth rate between subgroups**

**Supplemental Table 1 Baseline characteristics between subgroups**

| **Variable** | **Thyroid Cancer Group**  **(n = 61)** | **Control Group (n = 183)** | ***p*** | **Cervical Lesions Group**  **(n = 112)** | **Control Group (n = 336)** | ***p*** |
| --- | --- | --- | --- | --- | --- | --- |
| **Age (years), M (Q₁, Q₃)** | 33.0 (29.0–36.0) | 33.0 (30.0–36.0) | 0.496 | 33.0 (30.0–35.3) | 32.0 (29.8–36.0) | 0.513 |
| **BMI (kg/m²), M (Q₁, Q₃)** | 21.72 (20.13–24.56) | 21.83 (20.20–24.00) | 0.807 | 21.81 (20.20–23.21) | 21.47 (19.92–23.41) | 0.537 |
| **bFSH (mIU/mL), M (Q₁, Q₃)** | 7.23 (6.22–8.17) | 7.44 (6.09–8.91) | 0.364 | 7.37 (6.18–8.92) | 7.22 (6.19–8.76) | 0.822 |
| **AFC (n), M (Q₁, Q₃)** | 12.0 (7.0–17.0) | 10.0 (7.0–17.0) | 0.571 | 8.5 (5.0–12.0) | 12.0 (7.0–17.0) | **<0.001**^*^ |
| **AMH (ng/mL), M (Q₁, Q₃)** | 3.24 (1.59–5.21) | 3.38 (1.75–5.77) | 0.741 | 2.48 (1.28–4.29) | 3.48 (1.80–5.99) | **<0.001**^*^ |
| **Infertility duration (years), M (Q₁, Q₃)** | 2.0 (1.0–4.0) | 2.0 (1.0–4.0) | 0.633 | 2.0 (1.0–4.0) | 3.0 (2.0–4.0) | 0.159 |
| **Infertility type, n(%)** |  |  | 0.874 |  |  | 0.956 |
| Primary | 41 (67.21%) | 125 (68.31%) |  | 56 (50.00%) | 169 (50.30%) |  |
| Secondary | 20 (32.79%) | 58 (31.69%) |  | 56 (50.00%) | 167 (49.70%) |  |
| **Infertility etiology, n(%)** |  |  |  |  |  |  |
| Ovarian insufficiency | 21 (34.43) | 64 (34.97) | 0.938 | 54 (48.21) | 111 (33.04) | **0.004**^*^ |
| Ovulatory disorders | 8 (13.11) | 9 (4.92) | 0.059 | 3 (2.68) | 14 (4.17) | 0.668 |
| Tubal/pelvic factors | 21 (34.43) | 82 (44.81) | 0.155 | 57 (50.89) | 174 (51.79) | 0.870 |
| Uterine factors | 8 (13.11) | 46 (25.14) | 0.050 | 21 (18.75) | 74 (22.02) | 0.463 |
| Endometriosis | 9 (14.75) | 25 (13.66) | 0.831 | 17 (15.18) | 33 (9.82) | 0.119 |
| Male factors | 17 (27.87) | 55 (30.05) | 0.746 | 33 (29.46) | 81 (24.11) | 0.260 |
| Unexplained | 8 (13.11) | 14 (7.65) | 0.197 | 11 (9.82) | 23 (6.85) | 0.303 |

Values are presented as median (25th percentile, 75th percentile) or proportion (%).

^*^*p*<0.05

Abbreviations: BMI, body mass index; bFSH, basal follicle stimulating hormone;AFC, antral follicle count; AMH, anti-Müllerian hormone;

**Supplemental Table 2 COH Outcomes and embryo transfer data between subgroups**

| **Variable** | **Thyroid Cancer Group**  **(n = 61)** | **Control Group**  **(n = 183)** | ***p*** | **Cervical Lesions Group**  **(n = 112)** | **Control Group**  **(n = 336)** | ***p*** |
| --- | --- | --- | --- | --- | --- | --- |
| **COH cycles** | 85 | 211 | — | 158 | 397 | — |
| **Gn days (days), M (Q₁, Q₃)** | 9.0 (8.0–11.0) | 10.0 (8.0–11.0) | 0.243 | 9.5 (8.0–11.0) | 10.0 (9.0–11.0) | 0.255 |
| **Total Gn dose (IU), M (Q₁, Q₃)** | 2287.50 (1800.00–3000.00) | 2400.00 (1950.00–3000.00) | 0.361 | 2325.00 (1516.88–3000.00) | 2325.00 (1777.50–2915.62) | 0.217 |
| **Follicles ≥14 mm on hCG day, M (Q₁, Q₃)** | 8.0 (4.0–11.0) | 8.0 (6.0–12.0) | 0.208 | 7.0 (4.0–11.0) | 9.0 (5.0–12.0) | **0.002**^*^ |
| **E2 on hCG day (pg/ml), M (Q₁, Q₃)** | 1669.00 (928.00–2547.00) | 1973.00 (1269.25–3205.75) | **0.018**^*^ | 1543.50 (968.00–2720.00) | 2096.00 (1320.50–3343.50) | **<0.001**^*^ |
| **COH protocol, n(%)** |  |  | 0.094 |  |  | **0.025**^*^ |
| GnRH agonist | 26 (30.59) | 96 (45.50) |  | 60 (37.97) | 189 (47.61) |  |
| GnRH antagonist | 41 (48.24) | 80 (37.91) |  | 58 (36.71%) | 149 (37.53) |  |
| PPOS | 17 (20.00) | 31 (14.69) |  | 6 (3.80) | 8 (2.02) |  |
| Others | 1 (1.18) | 4 (1.90) |  | 34 (21.52) | 51 (12.85) |  |
| **Fertilization method, n (%)** |  |  | **0.006**^*^ |  |  | 0.531 |
| IVF | 47 (55.95) | 151 (72.60) |  | 115 (75.16) | 285 (72.52) |  |
| ICSI | 37 (44.05) | 57 (27.40) |  | 38 (24.84) | 108 (27.48) |  |
| **Total oocytes retrieved, M (Q₁, Q₃)** | 10.0 (6.0–17.0) | 9.0 (6.0–15.0) | 0.299 | 8.0 (4.0–14.0) | 10.0 (6.0–15.0) | **0.024**^*^ |
| **MII oocytes, M (Q₁, Q₃)** | 9.0 (6.0–13.0) | 8.0 (5.0–13.0) | 0.282 | 7.5 (4.0–11.8) | 8.0 (5.0–13.0) | **0.049**^*^ |
| **2PN embryos, M (Q₁, Q₃)** | 6.0 (3.0–9.0) | 6.0 (3.0–9.0) | 0.802 | 5.0 (2.0–8.0) | 6.0 (3.0–10.0) | **0.015**^*^ |
| **Fertilization rate, % (n)** | 56.44 (565/1001) | 59.42 (1340/2255) | 0.111 | 59.83 (901/1506) | 59.15 (2586/4372) | 0.644 |
| **Blastocyst formation rate, % (n)** | 61.10 (278/455) | 66.01 (738/1118) | 0.065 | 64.90 (466/718) | 67.17 (1516/2257) | 0.262 |
| **Available embryos, M (Q₁, Q₃)** | 3.0 (2.0–5.0) | 3.0 (2.0–5.0) | 0.225 | 3.0 (1.0–4.0) | 3.0 (2.0–5.0) | **0.002**^*^ |
| **Transfer cycles** | 93 | 291 | — | 207 | 520 | — |
| **Transfer cycle type, % (n)** |  |  | **0.007**^*^ |  |  | **0.007**^*^ |
| Fresh cycles only | 21.18 (18/85) | 29.86 (63/211) |  | 19.62 (31/158) | 30.73 (122/397) |  |
| Thawed cycles only | 47.06 (40/85) | 40.28 (85/211) |  | 44.30 (70/158) | 41.06 (163/397) |  |
| Fresh + thawed cycles | 10.59 (9/85) | 20.38 (43/211) |  | 18.35 (29/158) | 18.89 (75/397) |  |
| No transfer | 21.18 (18/85) | 9.48 (20/211) |  | 17.72(28/158) | 9.32 (37/397) |  |
| **Embryos transferred** | 116 | 375 | — | 258 | 654 | — |
| **Embryo type transferred, % (n)** |  |  | 0.136 |  |  | 0.396 |
| Cleavage-stage | 45.69 (53/116) | 53.60 (201/375) |  | 51.94 (134/258) | 55.05 (360/654) |  |
| Blastocysts | 54.31 (63/116) | 46.40 (174/375) |  | 48.06 (124/258) | 44.95 (294/654) |  |
| **Number of embryos transferred, % (n)** |  |  | 0.439 |  |  | 0.752 |
| Single | 74.82 (70/93) | 71.13 (207/291) |  | 75.36 (156/207) | 74.23 (386/520) |  |
| Double | 25.18 (23/93) | 28.87 (84/291) |  | 24.64 (51/207) | 25.77 (134/520) |  |
| **Endometrial preparation for thawed cycles, % (n)** |  |  | 0.390 |  |  | 0.327 |
| Artificial cycle | 69.70 (46/66) | 72.43 (134/185) |  | 70.07 (103/147) | 73.07 (236/323) |  |
| Natural cycle | 9.09 (6/66) | 12.43 (23/185) |  | 7.48 (11/147) | 9.91 (32/323) |  |
| Down-regulated + artificial cycle | 15.15 (10/66) | 12.97 (24/185) |  | 21.09 (31/147) | 14.86 (48/323) |  |
| Stimulated cycle | 6.06 (4/66) | 2.16 (4/185) |  | 1.36 (2/147) | 2.17 (7/323) |  |

Values are presented as median (25th percentile, 75th percentile) or proportion (%).

^*^*p*<0.05

Abbreviations: COH, controlled ovarian hyperstimulation; Gn, gonadotrophin; hCG, human chorionic gonadotrophin; PPOS, progestin-primed ovarian stimulation; IVF, *in vitro* fertilization; ICSI, intracytoplasmic sperm injection; MⅡ, metaphase II; 2PN, double pronucleus.

**Supplemental Table 3 Pregnancy and maternal-fetal outcomes between subgroups**

| **Variable** | **Thyroid Cancer Group**  **(n = 61)** | **Control Group**  **(n = 183)** | ***p*** | **Cervical Lesions Group**  **(n = 112)** | **Control Group (n = 336)** | ***p*** |
| --- | --- | --- | --- | --- | --- | --- |
| **Pregnancy outcomes, % (n)** |  |  |  |  |  |  |
| hCG positivity rate | 56.99 (53/93) | 51.20 (149/291) | 0.331 | 60.39 (125/207) | 53.85 (280/520) | 0.109 |
| Clinical pregnancy rate | 51.61 (48/93) | 47.08 (137/291) | 0.446 | 34.78 (72/207) | 48.08 (250/520) | **0.001**^*^ |
| Miscarriage rate | 22.91 (11/48) | 20.74 (28/137) | 0.717 | 25.00 (18/72) | 18.00 (45/250) | 0.187 |
| Ectopic pregnancy rate | 1.08 (1/93) | 0 | 0.242 | 0.48 (1/207) | 0 | 0.285 |
| Live birth rate | 39.78 (37/93) | 37.46 (109/291) | 0.687 | 26.09 (54/207) | 39.42 (205/520) | **<0.001**^*^ |
| **Maternal-fetal outcomes** |  |  |  |  |  |  |
| Number of neonates | 38 | 120 | — | 56 | 225 | — |
| **Fetal number, % (n)** |  |  | 0.765 |  |  | 0.252 |
| Singleton | 91.85 (1/37) | 91.03 (11/109) |  | 96.30 (52/54) | 90.24 (185/225) |  |
| Twin | 8.15 (36/37) | 8.97 (98/109) |  | 3.70 (2/54) | 9.76 (20/225) |  |
| **Delivery mode, % (n)** |  |  | 0.756 |  |  | 0.336 |
| Vaginal delivery | 21.62 (8/37) | 19.27 (21/109) |  | 12.96 (7/54) | 18.54 (38/205) |  |
| Cesarean section | 78.38 (29/37) | 80.73 (88/109) |  | 87.04 (47/54) | 81.46 (167/205) |  |
| **Gestational age (weeks), M (Q₁, Q₃)** | 38.8 (37.5–39.6) | 38.3 (37.9–39.5) | 0.724 | 38.2 (36.8–39.1) | 38.9 (37.9–39.5) | **0.014**^*^ |
| **Neonatal weight (kg), M (Q₁, Q₃)** | 3.30 (2.90–3.50) | 3.20 (2.74–3.56) | 0.501 | 3.21 (2.56–3.51) | 3.20 (2.90–3.50) | 0.704 |
| **Macrosomia, % (n)** | 0 | 1.67 (2/120) | 1.000 | 7.14 (4/56) | 2.22 (5/225) | 0.148 |
| **Low birth weight, % (n)** | 7.89 (3/93) | 15.83 (19/120) | 0.218 | 28.57 (16/56) | 15.11 (34/225) | **0.018**^*^ |
| **Preterm birth, % (n)** | 13.51 (5/37) | 12.84 (14/109) | 1.000 | 27.78 (15/54) | 15.61 (32/205) | **0.039**^*^ |
| **Gestational hypertension, % (n)** | 2.70 (1/37) | 2.75 (3/109) | 1.000 | 1.85 (1/54) | 2.44 (5/205) | 1.000 |
| **Gestational diabetes, % (n)** | 2.70 (1/37) | 1.83 (2/109) | 1.000 | 3.70 (2/54) | 4.39 (9/205) | 1.000 |
| **Placenta previa, % (n)** | 2.70 (1/37) | 1.83 (2/109) | 1.000 | 5.56 (3/54) | 1.95 (4/205) | 0.326 |
| **Premature rupture of membranes, % (n)** | 0 | 0.92 (1/109) | 1.000 | 1.85 (1/54) | 1.95 (4/205) | 1.000 |
| **Neonatal jaundice, % (n)** | 2.63 (1/38) | 4.17 (5/120) | 1.000 | 8.93 (5/56) | 5.78 (13/225) | 0.578 |
| **Neonatal birth defects, % (n)** | 5.26 (2/38) | 0.83 (1/120) | 0.144 | 3.57 (2/56) | 1.78 (4/225) | 0.753 |

Values are presented as median (25th percentile, 75th percentile) or proportion (%).

^*^*p*<0.05

Abbreviations: hCG, human chorionic gonadotrophin.

**Supplemental Table 4 Multi-cycle pregnancy outcomes between subgroups**

| **Group** | **Number of Patients** | **Oocyte Retrieval Cycles** | **Live Births** | **Single-Cycle CLBR (%)** | **Multi-Cycle CLBR** | |
| --- | --- | --- | --- | --- | --- | --- |
|  |  |  |  |  | **Conservative CLBR %**  **(95% CI)** | **Optimistic CLBR % (95% CI)** |
| **Thyroid Cancer Group** |  |  |  |  |  |  |
| Cycle 1 | 61 | 61 | 32 | 52.5 | 52.5 (39.9–65.0) | 52.5 (39.9–65.0) |
| Cycle 2 | 13 | 13 | 4 | 30.8 | 59.0 (46.7–71.4) | 67.2 (55.4–78.9) |
| Cycle 3 | 6 | 5 | 1 | 16.7 | 60.7 (48.4–72.9) | 72.6 (61.4–83.8) |
| ≥4 Cycles | 5 | 5 | 0 | 0.0 | 60.7 (48.4–72.9) | 72.6 (61.4–83.8) |
| **Control Group** |  |  |  |  |  |  |
| Cycle 1 | 183 | 182 | 102 | 55.7 | 55.7 (48.5–62.9) | 55.7 (48.5–62.9) |
| Cycle 2 | 21 | 20 | 6 | 28.6 | 59.0 (51.9–66.1) | 68.3 (61.6–75.0) |
| Cycle 3 | 5 | 4 | 1 | 20.0 | 59.6 (52.5–66.7) | 74.8 (68.6–81.1) |
| ≥4 Cycles | 2 | 2 | 0 | 0.0 | 59.6 (52.5–66.7) | 74.8 (68.6–81.1) |
| **Cervical Lesion Group** |  |  |  |  |  |  |
| Cycle 1 | 112 | 108 | 43 | 38.4 | 38.4 (29.4–47.4) | 38.4 (29.4–47.4) |
| Cycle 2 | 32 | 31 | 9 | 28.1 | 46.4 (37.2–55.7) | 55.7 (46.5–64.9) |
| Cycle 3 | 12 | 12 | 2 | 16.7 | 48.2 (39.0–57.5) | 63.1 (54.2–72.0) |
| ≥4 Cycles | 2 | 2 | 0 | 0.0 | 48.2 (39.0–57.5) | 63.1 (54.2–72.0) |
| **Control Group** |  |  |  |  |  |  |
| Cycle 1 | 336 | 335 | 196 | 58.3 | 58.3 (53.1–63.6) | 58.3 (53.1–63.6) |
| Cycle 2 | 42 | 40 | 8 | 19.1 | 60.7 (55.5–65.9) | 66.3 (61.3–71.4) |
| Cycle 3 | 10 | 9 | 1 | 10.0 | 61.0 (55.8–66.2) | 69.6 (64.7–74.6) |
| ≥4 Cycles | 9 | 9 | 0 | 0.0 | 61.0 (55.8–66.2) | 69.6 (64.7–74.6) |

Abbreviations: CLBR, cumulative live birth rate;CI, confidence interval
